# A Phenotyping Platform to Characterize Healthy Individuals Across Four Stages of Life - The *Enable* Study

**DOI:** 10.3389/fnut.2020.582387

**Published:** 2020-10-28

**Authors:** Beate Brandl, Thomas Skurk, Rachel Rennekamp, Anne Hannink, Eva Kiesswetter, Jessica Freiherr, Susanne Ihsen, Jutta Roosen, Martin Klingenspor, Dirk Haller, Dietmar Krautwurst, Thomas Hofmann, Jakob Linseisen, Dorothee Volkert, Hans Hauner

**Affiliations:** ^1^ZIEL - Institute for Food & Health, Technical University of Munich, Freising, Germany; ^2^Nutritional Medicine, Else Kroener-Fresenius-Centre for Nutritional Medicine, School of Life Sciences, Technical University of Munich, Freising, Germany; ^3^Institute for Biomedicine of Aging, Friedrich-Alexander-University Erlangen-Nürnberg, Nuremberg, Germany; ^4^Section of Neuroscience of Sensory Perception, Department of Psychiatry and Psychotherapy, Friedrich-Alexander-University Erlangen-Nürnberg, Erlangen, Germany; ^5^Department Sensory Analytics, Fraunhofer Institute for Process Engineering and Packaging (IVV), Freising, Germany; ^6^Gender Studies in Science and Engineering, Technical University of Munich, Munich, Germany; ^7^Marketing and Consumer Research, TUM School of Management, Technical University of Munich, Freising, Germany; ^8^Molecular Nutritional Medicine, TUM School of Life Sciences Weihenstephan, Technical University of Munich, Freising, Germany; ^9^Nutrition and Immunology, Technical University of Munich, Munich, Germany; ^10^Leibniz-Institute for Food Systems Biology at the Technical University of Munich, Freising, Germany; ^11^Food Chemistry and Molecular Sensory Science, Technical University of Munich, Munich, Germany; ^12^Helmholtz Zentrum Munich, German Research Center for Environmental Health, Independent Research Group Clinical Epidemiology, Neuherberg, Germany; ^13^Epidemiology, LMU Munich, UNIKA-T, Augsburg, Germany; ^14^School of Medicine, Institute of Nutritional Medicine, Klinikum Rechts der Isar, Technical University of Munich, Munich, Germany

**Keywords:** metabolic phenotyping, nutrition, *enable*-cluster, cohort, biosamples

## Abstract

**Introduction:** Nutritional habits and requirements are changing over the lifespan, but the dynamics of nutritional issues and the diet-health relationship in the major stages of the human life cycle are not sufficiently understood. A human phenotyping research platform for nutrition studies was established to recruit and phenotype selected population groups across different stages of life. The project is the backbone of the highly interdisciplinary *enable* competence cluster of nutrition research aiming to identify dietary determinants of a healthy life throughout the lifespan and to develop healthier and tasty convenience foods with high consumer acceptance.

**Methods:** The phenotyping program included anthropometry, body composition analysis, assessment of energy metabolism, health and functional status, multisensory perception, metabolic phenotyping, lifestyle, sociodemography, chronobiology, and assessment of dietary intake including food preferences and aversions.

**Results:** In total, 503 healthy volunteers at four defined phases of life including 3–5-year old children (*n* = 44), young adults aged 18–25 years (*n* = 94), adults aged 40–65 years (“middle agers,” *n* = 205), and older adults aged 75–85 years (*n* = 160) were recruited and comprehensively phenotyped. Plasma, serum, buffy coat, urine, feces and saliva samples were collected and stored at −80°C. Significant differences in anthropometric and metabolic parameters between the four groups were found. A major finding was the decrease in fat-free mass and the concomitant increase in % body fat in both sexes across the adult lifespan.

**Conclusions:** The dataset will provide novel information on differences in diet-related parameters over the lifespan and is available for targeted analyses. We expect that this novel platform approach will have implications for the development of innovative food products tailored to promote healthy eating throughout life.

**Trial registration:** DRKS, DRKS00009797. Registered on 20 January 2016, https://www.drks.de/drks_web/navigate.do?navigationId=trial.HTML&_ID=DRKS00009797.

## Introduction

Dietary habits have substantial impact on the onset and course of many chronic diseases (1). Nutrition behavior, however, is subject to multiple influences such as age, sociodemographic factors, and sensory food preferences among many others (2, 3). Although only limited knowledge about specific nutrient requirements and food selection in dependence of life stage is available, substantial differences in food selection and consumption may exist due to many complex interactions and temporal changes. Whereas food selection in young children may depend on parents' nutrition attitudes (4), young adults leaving their familial environment may develop their own lifestyle under strong peer-group influence (5, 6). Middle-aged adults following a Western dietary pattern and a sedentary lifestyle are at high risk of developing lifestyle-associated chronic diseases. After retirement, dietary requirements and habits may differ from younger age groups with a focus on the relief of physical impairments (7).

During these developmental stages dietary habits play a substantial role for subjective well-being and the objective health status. Of central importance, the progression of caloric overnutrition owing to energy dense foods and other external adverse influences begins already early in life (8). Furthermore, due to the increasing number of old and very old people, prevention of age-related chronic diseases and sustained independence in daily life are of utmost individual and public health interest (9). Thus, the challenge to improve dietary habits in a given age group covers a broad range of specific aspects. It is obvious that each life stage has its own needs, but the dynamics of food selection in the major stages of the human life cycle are not sufficiently understood (10, 11). It was, therefore, the rationale of this project to establish a unique phenotyping platform combining expertise from different disciplines, such as nutrition science, sensory science and consumer science among many others to get a better understanding of the diet-health relationship by a deep and comprehensive characterization of individuals in defined stages of life.

Thus, the central goal of the project is to characterize the dietary requirements and preferences and their relationship to health parameters in defined age groups. This knowledge may facilitate the development of age-group specific and, eventually, personalized dietary strategies for innovative health promotion and disease prevention. In this context, an additional goal was to establish a common databank and biobank that can be used for answering many associated research questions and which is accessible for external collaborators.

## Materials and Methods

### Ethics Statement

The study protocol was approved by the ethical committee of the Faculty of Medicine of the Technical University of Munich in Germany (approval no. 452/15) and Friedrich-Alexander-Universität Erlangen-Nürnberg (approval no. 291/15B). The guidelines of the International Conference on Harmonization of Good Clinical Practice and the World Medical Association Declaration of Helsinki (in the revised version of Seoul, South Korea 2008) were considered. All study participants have given written informed consent.

### Study Design and Setting

This cross-sectional study was performed between February 2016 and February 2018 in two centers in Freising and Nuremberg, Germany. A random sample of volunteers from the general population was recruited by advertising in kindergarten, newspapers and other media in the region of Freising and Nuremberg, Germany. Aim and procedures were explained during a presentation of the scientific staff at information meetings.

### Participant Inclusion/Exclusion Criteria

**Figure 1:**
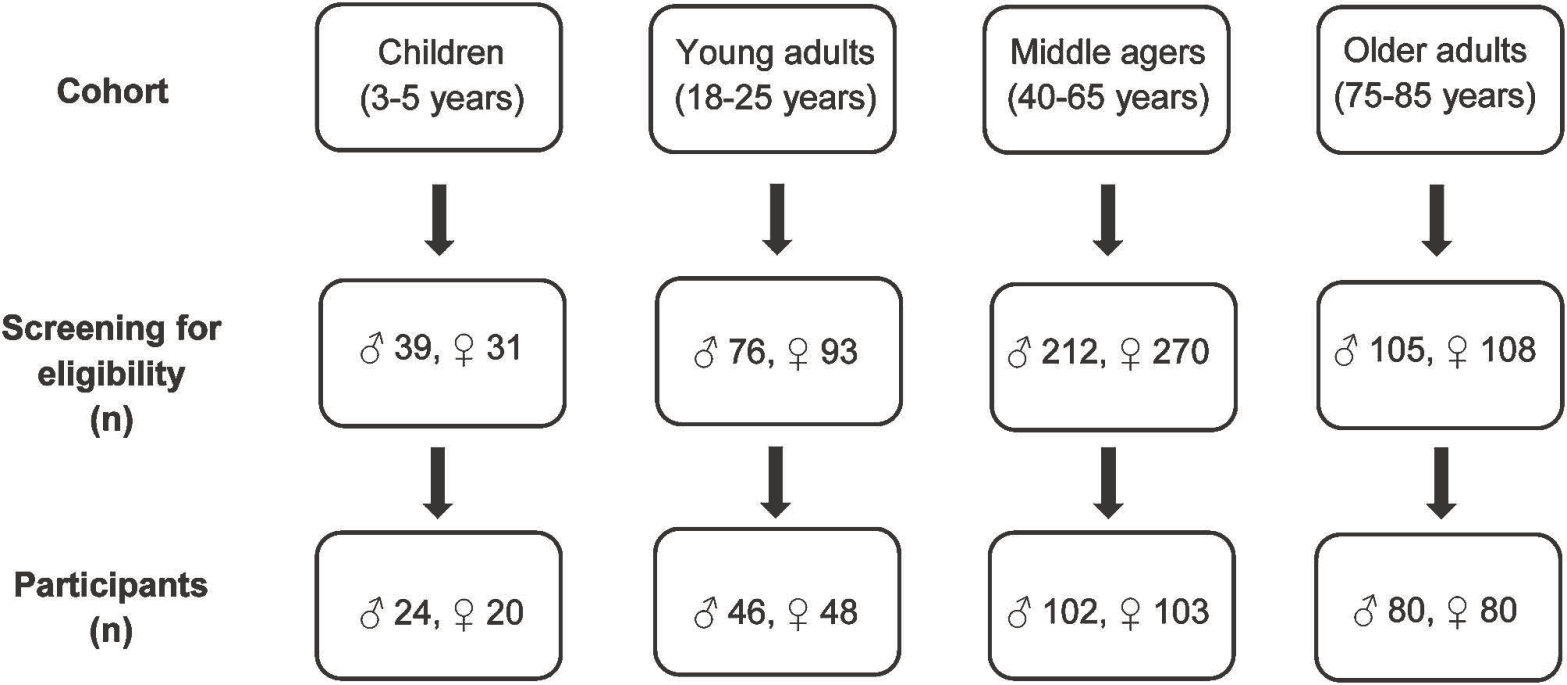
Flowchart of the recruitment process in the four age groups.

The eligibility of the volunteers was assessed using a detailed screening procedure either face-to-face or via telephone. Furthermore, an equal gender partition was planned for each age group. Moreover, in the age group 40–65 years half of the participants were selected with an elevated waist circumference (≥ 102 cm in males and ≥ 88 cm for females) indicating a moderately elevated cardiovascular and metabolic risk (Figure 1).

General inclusion criteria were healthy non-smoking community-dwelling Caucasians, body mass index (BMI) 18.5–30.0 (−35.0) kg/m^2^, Mini Mental State Examination (MMSE) ≥ 24 points (12) (the latter in older adults only). Explicit exclusion criteria in the children's group were chronic diseases such as asthma, endocrine diseases, and genetic syndromes such as trisomy 21. In the group of young adults and middle agers the following exclusion criteria were considered: pregnancy, chronic infections such as human immunodeficiency virus infection, endocrine diseases such as diabetes mellitus, history of myocardial infarction or stroke, untreated hypertension (>160/95 mmHg), heart failure, any reported cancer within the last 3 years, severe lung, liver, kidney disease, autoimmune disease, stomach ulcer, diagnosed psychological or neurological disease, blood transfusion in the last 3 months, immobility, unintended or intentional weight loss of more than 5% in the previous 3 months, and participation in intervention studies. In the age group 75–85 years, the same exclusion criteria were applicable as for the young adults and the middle-aged group with the addition: need of care (classification into a care level according to SGB §XI).

Finally, the four age groups included 44 3- to 5-year old children, 94 young adults aged 18–25 years, 205 adults aged 40–65 years (“middle agers”) and 160 older persons aged 75–85 years. In the group of middle agers 97 subjects were at risk for cardiometabolic disease defined by a waist circumference of ≥ 102 cm in males and ≥ 88 cm in females.

### Phenotyping of the Participants

**Figure 2:**
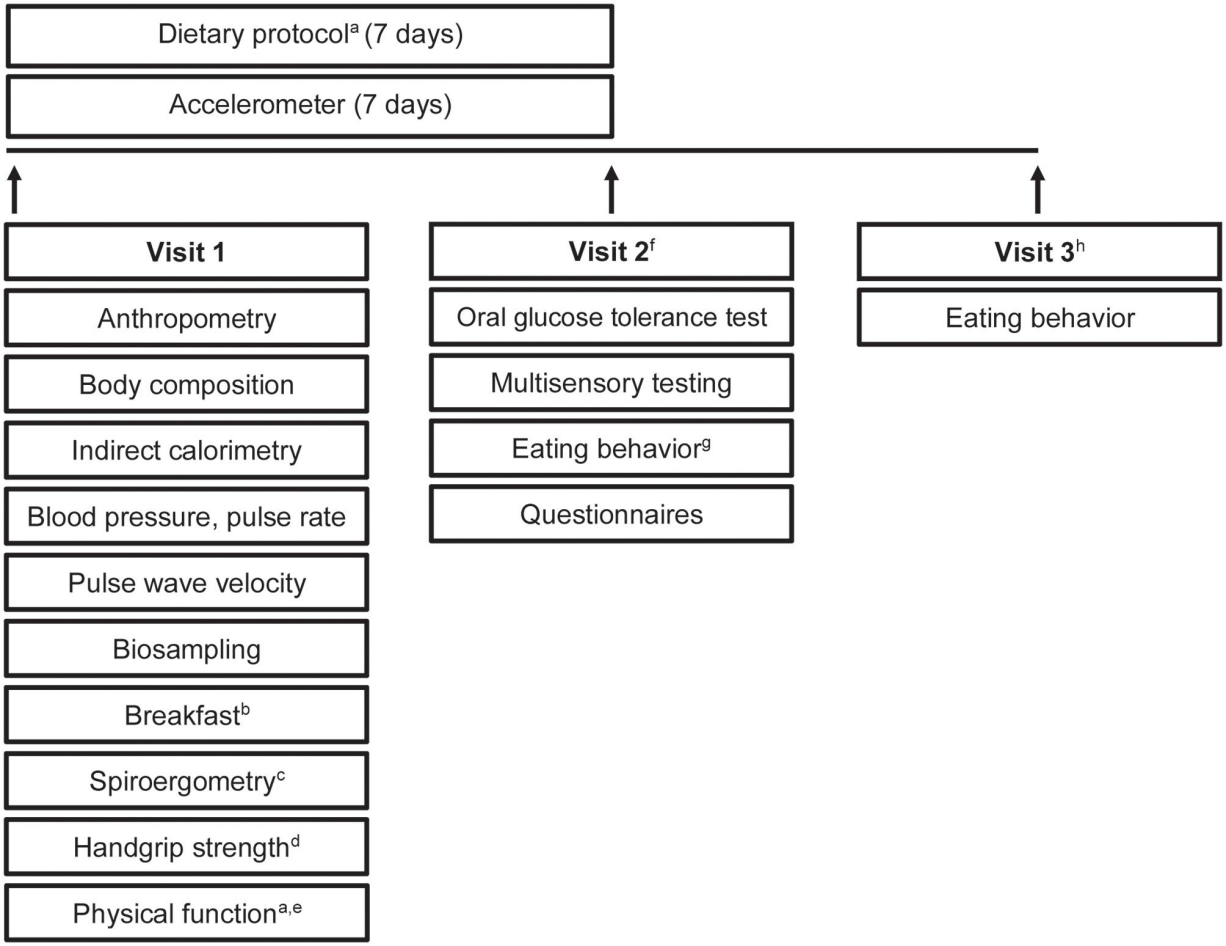
Study design. Timeline and examinations. a, only in older participants; b, standardized breakfast; c, only in young adults and middle agers; d, not in children; e, physical function included tests of the Short Physical Performance Battery and Fullerton Senior Fitness Test Battery; f, young adults, middle agers, older adults were invited for visit 2; g, eating habits were recorded by using a food frequency questionnaire and a 24-h food list; h, visit 3 included a web-based 24-h food list which could be filled at home.

All participants underwent a comprehensive phenotyping program (Figure 2). An overview of measurements and questionnaires in the respective age group is depicted in Tables 1, 2. All measurements and sampling processes were performed using established standard operation procedures (SOPs) by trained personnel.

**Table 1:** Overview of examinations in the four age cohorts.

	**Children (3–5 years)**	**Young adults (18–25 years)**	**Middle** **agers** **(40–65** **years)**	**Older** **adults** **(75–85** **years)**
**ANTHROPOMETRY AND BODY COMPOSITION**				
Body height	✓	✓	✓	✓
Body weight	✓	✓	✓	✓
Waist circumference	✓	✓	✓	✓
Hip circumference	✓	✓	✓	✓
Skin fold thickness	✓	–	–	–
Head circumference	✓	–	–	–
Upper arm circumference	–	–	–	✓
Calf circumference	–	–	–	✓
Bioelectrical impedance analysis	–	✓	✓	✓
Air Displacement Plethysmography	✓	✓	✓	✓^a^
**ENERGY METABOLISM**				
Indirect calorimetry	✓	✓	✓	✓
Accelerometer	✓	✓	✓	✓
**HEALTH AND FUNCTIONAL STATUS**				
Blood pressure, pulse rate	✓	✓	✓	✓
Pulse wave velocity	✓	✓	✓	✓^a^
Spiroergometry	–	✓	✓	–
Physical function	–	–	–	✓
Handgrip strength	–	✓	✓	✓
**MULTISENSORY TESTING**				
Visual function	–	✓	✓	✓
Hearing ability	–	✓	✓	✓
Olfaction test	✓	✓	✓	✓
**METABOLISM**				
Oral glucose tolerance test	–	✓	✓	✓
**BIOSAMPLING**				
Plasma	–	✓	✓	✓
Serum	–	✓	✓	✓
Dried Blood Spots	–	✓	✓	✓
Urine	✓	✓	✓	✓
Feces	✓	✓	✓	✓
Genetic material	Saliva	Buffy coat	Buffy coat	Buffy coat

a *Measurement was only performed in Freising, Germany (all children, young adults, middle-agers and 54 seniors)*.

**Table 2:** Overview of all questionnaires in four age cohorts.

	**Children (3–5 years)**	**Young adults (18–25 years)**	**Middle agers (40–65 years)**	**Older** **adults (75–85 years)**
**HEALTH STATUS**				
Birth weight	✓	–	–	–
Subjective health	✓	✓	✓	✓
Physical well-being	✓	–	–	–
Mental well-being	✓	–	–	–
Self-worth	✓	–	–	–
Satisfaction with live/health	–	✓	✓	✓
Body weight and weight changes	–	✓	✓	✓
Disease	–	✓	✓	✓
Pain	–	–	–	✓
Functional limitations (LLFDI)	–	–	–	✓
Falls, fear of falling	–	–	–	✓
**LIFESTYLE**				
Smoking	–	✓	✓	✓
Physical activity	✓	✓	✓	✓
Television/computer time	✓	✓	✓	✓
Sleeping duration	✓	–	–	–
**EATING BEHAVIOR**				
Breastfeeding	✓	✓	–	–
Meal habits and special diets	✓	✓	✓	✓
Appetite	–	✓	✓	✓
Nutritional effects of life events	–	✓	✓	✓
Knowledge	–	✓	✓	✓
Attitudes toward nutrition and health	–	✓	✓	✓
Food selection	–	✓	✓	✓
Eating motives (TEMS)	–	✓	✓	✓
Dietary protocol	–	–	–	✓
Food preferences and aversions	–	✓	✓	✓
Food frequency questionnaire	✓	✓	✓	✓
Two 24 h- food lists	✓	✓	✓	✓
**SOCIODEMOGRAPHIC**				
Supervisory relationships	✓	✓	–	–
Living conditions	✓	✓	✓	✓
Marital status	–	✓	✓	✓
Nationality, native language	✓	✓	✓	✓
Education	✓	✓	✓	✓
Professional qualification	✓	✓	✓	✓
Occupational status	✓	✓	✓	✓
Voluntary commitment	✓	✓	✓	✓
Financial status/ income	✓	✓	✓	✓
Social network	–	✓	✓	✓
**Chronobiology**	✓	✓	✓	✓

#### Anthropometry and Body Composition

All anthropometric and clinical parameters were measured in the morning following an overnight fast. In the group of 3–5-year-old participants, head circumference was determined. Furthermore, the skinfold thickness was measured at the triceps, subscapular, and suprailiacal location with a skinfold caliper (Holtain Ltd., Crosswell, UK). In the older persons, the upper arm circumference and calf circumference were additionally measured. Body composition and weight were measured using the Seca mBCA 515 device (Seca GmbH & Co KG, Hamburg, Germany). In addition, Bod Pod (COSMED, Fridolfing, Germany) was used to assess body composition (Freising only).

#### Energy Metabolism

Resting metabolic rate (RMR) was calculated based on gas exchange measurements performed by indirect calorimetry under a canopy hood (COSMED Indirect calorimetry, Fridolfing, Germany). Data acquisition was performed during 30 min under thermoneutral conditions. Furthermore, participants wore standard accelerometry devices to monitor their everyday physical activity for seven days all day long (ActiGraph wGT3X-BT, Pensacola, FL, USA). Recorded data was analyzed using ActiLife software (version 6.13.3; Acti-Graph Corp., Pensacola, FL).

#### Health and Functional Status

Systolic and diastolic blood pressure and pulse rate were assessed using Omron M8 comfort (Mannheim, Germany), or Maxi Stabil 3 (WelchAllyn GmbH &Co. KG; Hechingen, Germany). Pulse wave velocity (PWV) was measured using Arteriograph (TensioMed Ltd, Budapest, Hungary) with the corresponding TensioMed analysis software (version 3.0.0.4) (Freising only). Spiroergometry was performed with the MetaLyser 3B-R3 (CORTEX Biophysik GmbH, Leipzig, Germany) to measure ventilatory thresholds (VT1, VT2) and O_2peak_. The respiratory gases were measured during a physical ramp stress until exhaustion or up to 300 watts in the young adults and middle-aged groups. Physical function of the older adults was assessed by the Short Physical Performance Battery (SPPB) (balance, gait speed, chair-rise) (13, 14), and the Fullerton Senior Fitness Test Battery (30 s chair stand test, 30 s arm curl test, chair sit and reach test, back stretch test, 8-Foot Up-and-Go test, 6 min walk) (15, 16). Handgrip strength was measured in young adults, middle agers and older adults by using a hydraulic hand grip dynamometer (JAMAR Model 5030J1, Ludwig Bertram GmbH, Isenhagen, Germany).

#### Multisensory Testing

Visual functions were assessed using the Landolt ring test. Additionally, red-green color blindness was checked with the Ishihara test. Hearing ability was checked using a standard audiometer (MAICO ST 20, MAICO Diagnostics GmbH, Berlin Germany). To test olfactory performance, the 40-item Monell Extended Sniffin' Sticks Identification Test (MONEX-40) was used (17). Finally, participants had to rate six recombinants of chemically defined fragrances regarding their intensity and pleasantness.

#### Oral Glucose Tolerance Test

Following a 12-h overnight fast an oral glucose tolerance test (oGTT) was performed. After taking a baseline blood sample in the fasting state, volunteers received 75 g glucose in a volume of 300 ml water (Carl Roth GmbH & Co. KG, Karlsruhe, Germany). After 30, 60, 90, 120, 180, and 240 min blood was drawn and plasma glucose levels were determined (HemoCue Glucose 201+, plasma-calibrated, Ängelholm, Sweden).

#### Biosampling

The collection of biomaterials was an important part of the *enable* phenotyping activities. As summarized in Table 1, plasma, serum, dried blood spots, urine, and feces were collected in the volunteers from the different age groups. In the children, collection of blood was not possible, but saliva was stored for genetic analyses.

All blood samples were collected in the fasting state. Routine laboratory parameters were analyzed by a certified laboratory (SynLab; Munich, Germany). Additional blood samples were collected, immediately centrifuged (2.500 *g* for 10 min at 20°C) and subsequently stored in aliquots at −80°C for further analyses. Buffy coats were collected for DNA extraction. Dried blood spots were prepared by pipetting 75 μl whole EDTA-blood to the center of each circle without touching the filter paper (Whatman® 903 Protein Saver Card, Sigma-Aldrich Chemie GmbH, Taufkirchen, Germany). The filter cards were left 4 h to dry in a horizontal position protected from light. After drying, samples were bagged together with desiccant (Silica gel, Carl Roth, Karlsruhe, Germany) and stored at −20°C for later measurements. Urine was collected from all participants in the morning. Osmolarity was measured by using an automatic digital osmometer (OM 815, Vogel GmbH & Co. KG, Fernwald, Germany) and samples were aliquoted and stored at −80°C until analysis. Participants collected stool in two tubes, whereby only one tube contained 8 ml DNA stabilization buffer (Stratec Molecular GmbH, Berlin, Germany). Participants were instructed to bring the samples to the study center within 6 h for storage at −80°C. Children collected 1 ml saliva using a saliva collection kit (Oragene DNA, OG-500, DNA Genotek, Ottawa, Canada). Samples were also stored at −80°C.

#### Eating Behavior

In the four age groups, eating/dietary habits were recorded in a web-based manner by using a food frequency questionnaire (FFQ2) (18). Moreover, a 24-h food list to identify foods consumed during the previous 24 h was filled in twice with a lag time of 3 months (19). In addition, the older participants were instructed to record their food consumption for 7 days in an open diary. Energy content and nutrient composition of the diets were calculated using EBISpro software (EBISpro 2016, Willstätt-Legelshurst, Germany). Furthermore, meal habits, appetite (20, 21), life events (22), eating motives (23), and food preferences/aversions were assessed by specific and validated questionnaires.

#### Other Parameters Assessed by Questionnaires

Validated questionnaires to assess the health status (20, 24–29), lifestyle (26, 30), sociodemography (20, 29), and chronobiology (31, 32) were applied in all age groups. Table 2 summarizes the parameters assessed by the various questionnaires.

### Data Management

A good clinical practice compliant (GCP), validated study data bank with a remote data entry system (electronic Case Report Form, eCRF) was set up by a trained data manager from the Munich Study Centre (MSZ, Technical University of Munich) according to established SOPs. Consistency and plausibility were checked automatically by the system. During the study period, regular internal monitoring was performed to ascertain high quality of data entry.

### Statistical Data Analysis

Data were analyzed in the R programming environment. Results are presented as mean ± SD. *p*-values < 0.05 are regarded as statistically significant. The effect of different age groups was analyzed with a one-way ANOVA test for normally distributed data and a Kruskal-Wallis test in case of non-normal data. In each case a *post-hoc* test was performed to analyze all pairwise comparisons. The *post-hoc* tests were done with an appropriate adjustment for multiple testing. Moreover, in each cohort, differences between females and males were assessed by using Mann-Whitney U test.

## Results

Selected anthropometric and metabolic characteristics of the study participants in the four age groups are presented in Table 3. Additional detailed information about anthropometry, body composition and laboratory analysis between females and males in each group are shown in the Supplementary Tables 1, 4.

**Table 3:** Anthropometric and metabolic characteristics of study participants.

	**Children, 3–5** **years**	**Young adults, 18–25 years**	**Middle agers, 40–65 years**	**Older adults, 75–85** **years**
*n* (%)	44 (♂ 55, ♀ 45)	94 (♂ 51, ♀ 49)	108 (♂ 52, ♀ 48)	160 (♂ 51, ♀ 49)
Age, y	4.16 ± 0.89^a^	22.2 ± 1.97^b^	52.2 ± 6.60^c^	78.2 ± 2.75^d^
Height, cm	108.6 ± 9.27^a^	175.5 ± 9.72^b^	172.6 ± 9.30^b^	166.7 ± 9.13^c^
Body weight (SECA), kg	17.9 ± 3.69^a^	68.2 ± 11.70^b^	72.6 ± 13.0^b^	74.5 ± 13.4^b,c^
BMI, kg/m^2^		22.1 ± 2.54^a^	24.2 ± 2.95^b^	26.5 ± 3.96^c^
Waist circumference, cm	50.3 ± 4.63^a^	77.9 ± 8.05^b^	84.3 ± 10.40^c^	96.3 ± 13.5^d^
Hip circumference, cm	55.8 ± 4.78^a^	95.6 ± 6.00^b^	96.8 ± 6.53^b^	102.6 ± 8.53^c^
AST, U/l		22.0 ± 7.50	23.6 ± 8.17	23.9 ± 7.84
ALT, U/l		23.9 ± 10.70^a,b^	25.8 ± 11.3^a^	21.7 ± 8.27^b^
γGT, U/l		16.9 ± 11.00^a^	25.1 ± 22.10^b^	27.9 ± 25.90^b^
Cholesterol, mg/dl		179.3 ± 31.60^a^	224.4 ± 39.50^b^	220.9 ± 43.20^b^
Triglycerides, mg/dl		93.8 ± 40.28	99.8 ± 53.37	107.3 ± 43.55
HDL-cholesterol, mg/dl		60.9 ± 16.36	63.5 ± 17.20	64.1 ± 16.77
LDL-cholesterol, mg/dl		102.2 ± 26.79^a^	136.4 ± 37.98^b^	137.8 ± 38.52^b^
Fasting blood glucose, mg/dl		76.7 ± 5.77^a^	82.4 ± 7.94^b^	89.1 ± 9.42^c^
Fasting insulin, μU/ml		3.21 ± 1.82^a^	3.61 ± 3.13^a^	5.05 ± 4.03^b^
TSH, μU/ml		1.77 ± 0.88^a^	1.38 ± 0.73^b^	1.34 ± 1.31^b^

*Data are presented as mean ± standard deviation. Middle agers fulfilling our criteria for “cardiometabolic risk” were not taken into account. P-values < 0.05 were regarded as statistically significant. The effect of different age groups was analyzed with a one-way ANOVA test for normally distributed data and a Kruskal-Wallis test in case of non-normal data. In each case a post-hoc test was performed to analyze all pairwise comparisons. The post-hoc tests were done with an appropriate adjustment for multiple testing. Means in a row labeled with a different superscript differ significantly, P < 0.05. AST, aspartate aminotransferase; ALT, alanine aminotransferase; γGT, gamma-glutamyltransferase; HDL, high-density lipoprotein; LDL, low-density lipoprotein; ns, not significant; TSH, Thyroid-stimulating hormone*.

### Anthropometric and Metabolic Differences Between Defined Age Groups

**Figure 3:**
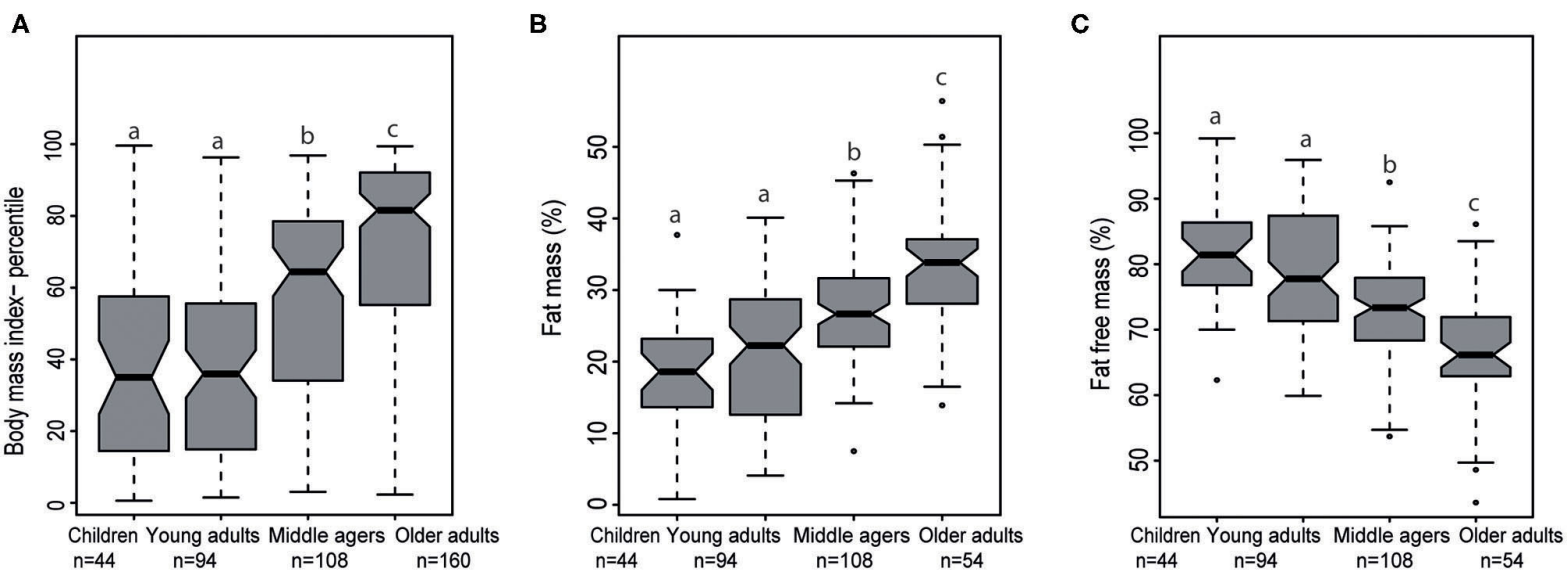
Notched Box plots of **(A)** body mass index percentile in the four stages of life, **(B)** fat mass (%), and **(C)** fat free mass (%) analyzed by air displacement plethysmography. Middle agers fulfilled our criteria for “cardiometabolic risk” were not taken into account. The effect of different age groups was analyzed with a one-way ANOVA test for normally distributed data and a Kruskal-Wallis test in case of non-normal data. In each case a *post-hoc* test was performed to analyze all pairwise comparisons. The *post-hoc* tests were done with an appropriate adjustment for multiple testing. Labeled means in a row without a common superscript letter differ, *P* < 0.05.

BMI percentile and percentage fat mass were increased significantly from young adults to middle agers up to older adults (each *p* < 0.001). As expected, percentage fat free mass significantly decreased from young adults to the middle agers and to the age group between 75 and 85 years (*p* < 0.05, Figures 3A–C).

Regarding liver enzymes, aspartate aminotransferase was not significantly different across the three adult age groups. Alanine aminotransferase was significantly lower in older adults compared to middle agers (*p* < 0.05). Gamma-glutamyltransferase levels were significantly lower in young adults compared to middle agers (*p* < 0.01) and to older adults (*p* < 0.05). Furthermore, young adults had a mean total cholesterol level of 179.3 ± 31.6 mg/dl that was significantly lower than in the middle-aged (224.4 ± 39.5 mg/dl) and in the older group (220.9 ± 43.2 mg/dl, each *p* < 0.05). Regarding triglycerides and HDL-cholesterol, no significant differences could be detected between the three age groups. In young adults, a mean LDL-cholesterol level of 102.2 ± 26.8 mg/dl was measured. In contrast, middle agers and older adults had significantly higher LDL-cholesterol levels (each *p* < 0.05). Fasting plasma glucose and insulin levels significantly increased across the three age groups, from 76.6 ± 5.8 mg/dl up to 89.1 ± 9.4 mg/dl, and from 3.2 ± 1.8 μU/ml to 5.1 ± 4.0 μU/ml, respectively (*p* < 0.05). In contrast, thyroid- stimulating hormone (TSH) levels were highest in the young adults with 1.8 ± 0.9 μU/ml and were significantly lower in middle agers and older adults, respectively (each *p* < 0.05).

### Anthropometry in the 3- to 5-Year Old Children

In the group of 3–5 years old children (*n* = 44, 24 boys, and 20 girls) there were no differences between both sexes in any of the anthropometric characteristics presented in Supplementary Table 1.

### Anthropometry and Metabolic Parameters in the Young Adults Aged 18–25 Years

Ninety-four young adults (48 males, 46 females) at a mean age of 22.2 ± 2.0 years participated in the study (Supplementary Table 2). All metabolic parameters were in the normal range, whereas female participants exhibited higher total cholesterol and HDL-cholesterol levels than male participants (*p* < 0.001). All fasting blood glucose levels were within the normal range (<100 mg/dl) and fasting insulin was higher in female than in male participants (*p* < 0.05).

### Anthropometry and Metabolic Parameters in the Middle Agers

In the group of middle agers, 108 volunteers (56 males, 52 females) with normal BMI (<25.0 kg/m^2^) participated (Supplementary Table 3). Two male participants had a fasting plasma glucose value beyond 100 mg/dl. Moreover, 81 of 108 participants (41 males, 40 females) had a total cholesterol level above 200 mg/dl.

Additionally, 97 participants (46 males, 51 females) of this age group fulfilled our criteria for “cardiometabolic risk” and had an elevated body weight (BMI > 25 kg/m^2^). Minor deviations from the normal ranges were more frequent than in the normal weight group as shown in Supplementary Table 3.

### Anthropometry and Metabolic Parameters in the Older Adults

One hundred-sixty community-dwelling participants (mean age 78.2 ± 2.8 years, mean BMI 26.5 ± 4.0 kg/m^2^) without physical and mental impairments were included (Table 3). Lipid abnormalities were frequently seen in this group. One hundred five participants had a total cholesterol level above 200 mg/dl. Sixteen (12 males, four females) of the 160 participants had a fasting plasma glucose beyond 100 mg/dl.

### Dietary Intake of Young Adults, Middle Agers, Older Adults

Dietary intake of participants was calculated based on FFQ2 and up to two 24 h food lists. Details are given elsewhere (33). Within young adults, middle agers, and older adults energy intake, fat intake (except unsaturated fatty acids), carbohydrate and protein intake did not differ significantly (Table 4). In contrast, fiber intakes in young adults was higher than in middle agers (*p* = 0.03).

**Table 4:** Dietary intake of young adults, middle agers, and older adults.

	**Young adults, 18–25 years**	**Middle agers, 40–65 years**	**Older adults, 75–85 years**
*n*	93	108	134
Energy intake, kcal/day	1,941 ± 387	1,937 ± 381	1,972 ± 421
Fat, g/day	81.4 ± 17.60	84.2 ± 16.43	84.6 ± 17.70
Fat, EN %	39.5	40.7	40.0
Saturated fatty acids, g/day	36.5 ± 8.36	37.1 ± 7.53	36.9 ± 7.53
Unsaturated fatty acids, g/day	28.0 ± 6.43^a^	30.1 ± 6.41^a,b^	30.5 ± 7.04^b^
Carbohydrates, g/day	209.7 ± 45.80	200.9 ± 45.42	211.4 ± 51.48
Carbohydrates, EN %	44.3	42.8	44.5
Fiber, g/day	22.7 ± 7.50^a^	20.5 ± 5.35^b^	22.1 ± 6.23^a,b^
Protein, g/day	76.7 ± 16.67	76.9 ± 15.52	73.8 ± 16.25
Protein, EN %	16.2	16.5	15.5

*Data are presented as mean ± standard deviation. P-values < 0.05 were regarded as statistically significant. Middle agers fulfilled our criteria for “cardiometabolic risk” were not taken into account. The effect of different age groups was analyzed with a one-way ANOVA test for normally distributed data and a Kruskal-Wallis test in case of non-normal data. In each case a post-hoc test was performed to analyze all pairwise comparisons. The post-hoc tests were done with an appropriate adjustment for multiple testing. Means in a row labeled with a different superscript differ significantly, P < 0.05. EN%, energy percent*.

## Discussion

The *enable* cluster represents a consortium of experts and institutions committed to create interdisciplinary collaborations focussing on food, nutrition, metabolism, energy balance and healthy aging. The cluster comprises academic research groups with expertise in the aforementioned fields together with consumer and social sciences, information and communication technologies, and food industry (http://www.enable-cluster.de).

As a unique feature and guiding principle, the *enable* cluster is addressing associations between nutrition behavior and quality and diet-related health issues in defined age groups, ranging from early childhood over young adulthood, middle age to older age, thereby covering life stages, in which individuals are exposed to different living conditions and environments. For this purpose, four defined age groups of healthy individuals were recruited and extensively phenotyped using highly standardized operating procedures. The major goal of the project is to compare determinants of food selection, eating behavior, and cardiometabolic disease risks across the four age groups in future analyses.

The current analysis contains a limited number of anthropometric, metabolic and dietary data across the four healthy age groups indicating how these parameters differ across age groups and sex. There is a clear trend toward an increase in percentage body fat mass and, in parallel, a decrease in percentage fat free mass in both sexes depending on the age group. The analysis of metabolic parameters showed age-dependent modest deteriorations particularly in glucose and lipid metabolism suggesting a slow decline in cardiometabolic health. It is unclear how dietary habits and patterns are accountable for these differences and if these alterations will require and benefit from modifications in dietary intake. Furthermore, hypotheses regarding the relationships between sensory qualities and eating behavior as well as metabolic health can be addressed. In the *enable* database potential links between gut microbiota, resting metabolic rate, and diet can be investigated in well-characterized participants and in different stages of life. In addition, within these age groups we are planning to link omics data (metabolomics, genetics and epigenetics) with clinical parameters, dietary patterns, or microbiota parameters to identify novel associations and to explore mechanistic links. More information on single research projects is available on the cluster web page (http://www.enable-cluster.de).

A strength of the project is that all assessments are based on defined SOPs and validated tools, e.g., questionnaires. A limitation may be that the groups were strictly defined to reduce heterogeneity. Due to the high selection bias, findings cannot be directly transferred to the general population. It is also important to note that the study is cross-sectional and does not allow conclusions on the development of certain parameters during lifetime, as the phenotypes in the specific age groups may have been influenced by former and no longer existing conditions. Irrespective of such limitations, the comparison of lifestyle and health characteristics across four age groups may provide valuable information for the development of age-specific recommendations for a health-promoting diet and lifestyle.

## Data Availability Statement

All datasets generated for this study are included in the article/Supplementary Material.

## Ethics Statement

The studies involving human participants were reviewed and approved by Faculty of Medicine of the Technical University of Munich in Germany. The patients/participants provided their written informed consent to participate in this study.

## Author Contributions

HH, DV, TS, JF, MK, and DH: conceived the experiments. HH, DV, TS, and BB: designed the phenotyping program. BB: performed study management and data management. BB, AH, and RR: were responsible for data collection and entry. JF: provided materials for sensory testing. TH, DK, and JF: were in charge of the chemosensory pheno-/genotyping to unravel food preference and aversion in the four age groups. BB, EK, DV, JR, SI, and JL: arranged the questionnaires. BB, TS, DV, and HH: wrote the manuscript. All authors critically read the manuscript and approved the final version.

## Conflict of Interest

The authors declare that the research was conducted in the absence of any commercial or financial relationships that could be construed as a potential conflict of interest.

## Abbreviations

eCRF: electronic Case Report Form
FFQ: Food Frequency Questionnaire
GCP: Good Clinical Practice
MetS: Metabolic Syndrome
MMSE: Mini-Mental State Examination
MONEX: Monell Extended Sniffin’ Sticks Identification Test
MSZ: Munich Study Centre
RMR: resting metabolic rate
SGB: social security statute book
SOP: standard operation procedure
SPPB: Short Physical Performance Battery
TSH: thyroid- stimulating hormone
VT: ventilatory threshold.
