# Supplementary material for: A Phenotyping Platform to Characterize Healthy Individuals Across Four Stages of Life - The Enable Study
Source: Front Nutr. 2020 Oct 28;7:582387. doi: 10.3389/fnut.2020.582387 (PMC7657309; doi:10.3389/fnut.2020.582387)
Supplement: Supplementary file 1 [file Data_Sheet_1.pdf]

Supplementary Material

Table 1. Study characteristics of 3- to 5-year old children.

	male	female	p-value
**n**	24	20	
**Anthropometrie**			
Age,y	4.38±0.82	3.9±0.91	ns
Height, cm	110.9±8.50	105.8±9.57	ns
Waist circumference, cm	50.8±4.83	49.6±4.41	ns
Hip circumference, cm	56.7±4.86	54.7±4.57	ns
Waist-to Hip ratio	0.90±0.06	0.91±0.04	ns
Brain circumference, cm	51.3±2.16	49.7±2.61	ns
Skin fold thickness, triceps, cm	0.82±0.29	0.84±0.35	ns
Skin fold thickness, subscapular, cm	0.89±1.11	0.66±0.33	ns
Skin fold thickness, suprailiacal, cm	0.71±0.40	0.69±0.37	ns
Body mass index-SDS	-0.31±1.11	-0.55±0.85	ns
Body mass index -percentile	41.9±26.9	33.6±26.4	ns
**Body composition**			
Seca, body weight, kg	18.9±3.59	16.7±3.54	ns
Bod Pod, Body weight, kg	18.9±3.76	16.9±3.92	ns
Bod Pod, Fat mass, kg	3.8±2.12	2.9±1.22	ns
Bod Pod, Fat free mass, kg	15.1±3.03	13.9±3.29	ns

Data are presented as mean ± standard deviation. *P*-values <0.05 were regarded as statistically significant. Mann-Whitney U test was used to test for significance between males and females in the cohort. ns, not significant.

Table 2. Study characteristics of young adults.

	male	female	p-value
**n**	48	46	
**Anthropometric**			
Age,y	22.3±2.1	22.0±1.85	ns
Height, cm	182.6±7.45	168.6±5.93	< 0.0001
Waist circumference, cm	80.8±8.81	75.1±6.14	< 0.001
Hip circumference, cm	95.4±6.42	95.9±5.63	ns
Waist-to Hip ratio	0.85±0.05	0.78±0.06	< 0.0001
BMI, kg/m²	22.7±2.99	21.5±1.86	ns
BMI - SDS	-0.36±0.97	-0.41±0.64	ns
BMI - percentile	39.5±28.7	36.6±21.6	ns
**Body composition**			
Seca			
Body weight, kg	75.7±11.4	61.1±6.31	< 0.0001
Fat mass, kg	12.2±7.22	15.9±3.67	< 0.0001
Fat free mass, kg	63.5±6.28	45.2±4.32	< 0.0001
Bod Pod			
Body weight, kg	75.7±11.3	61.1±6.31	< 0.0001
Fat mass, kg	11.6±6.84	16.8±4.22	< 0.0001
Fat free mass, kg	64.0±7.69	44.2±5.04	< 0.0001
**Laboratory analysis**			
AST, U/l	22.6±6.62	21.4±8.27	ns
ALT, U/l	27.8±9.89	20.2±10.2	< 0.0001
γGT, U/l	20.5±12.1	13.5±8.58	< 0.0001
Sodium, mmol/l	140.9±2.12	140.4±1.79	ns
Potassium, mmol/l	4.32±0.28	4.27±0.26	ns
Calcium, mmol/l	2.41±0.15	2.35±0.09	< 0.001
Phosphorus, mmol/l	3.49±0.39	3.88±0.39	< 0.0001
Magnesium, , mmol/l	0.87±0.04	0.85±0.05	< 0.05
Cholesterol, mg/dl	168.0±27.9	189.9±31.3	< 0.001
Triglycerides, mg/dl	92.8±44.8	94.8±35.9	ns
HDL-cholesterol, mg/dl	51.7±11.4	69.5±15.7	< 0.0001
LDL-cholesterol, mg/dl	97.2±25.4	106.8±27.5	ns

LDL/HDL	1.98±0.66	1.60±0.52	< 0.05
Creatinine, mg/dl	0.87±0.11	0.74±0.10	< 0.0001
Urea, mg/dl	29.2±5.88	24.7±5.14	< 0.001
Uric acid, mg/dl	5.74±0.95	4.51±1.06	< 0.0001
Fasting blood glucose, mg/dl	77.5±5.55	75.2±5.81	ns
Fasting insulin, µU/ml	2.94±1.89	3.47±1.73	< 0.05
TSH, µU/ml	1.92±0.84	1.63±0.89	ns
FT3, pg/ml	3.68±0.40	3.19±0.42	< 0.0001
FT4, ng/dl	1.23±0.16	1.08±0.14	< 0.0001

Data are presented as mean ± standard deviation. *P*-values <0.05 were regarded as statistically significant. Mann-Whitney U test was used to test for significance between female and male participants. AST, aspartate aminotransferase; ALT, alanine aminotransferase; BMI, body mass index; γGT, gamma glutamyltransferase; HDL, high- density lipoprotein; LDL, low-density lipoprotein; ns, not significant; TSH, thyroid-stimulating hormone; FT3, free thyroxine; FT4, free triiodothyronine.

Table 3. Characteristics of middle agers.

	Middle agers without cardiometabolic risk			Middle agers with cardiometabolic risk		
	**male**	**female**	**p-value**	**male**	**female**	**p-value**
**n**	56	52		46	51	
**Anthropometry**						
Age,y	52.1±7.1	52.2±6.05	ns	55.2±6.69	50.6±7.22	< 0.01
Height, cm	179.3±5.84	165.3±6.24	<0.0001	179.7±5.28	165.9±6.10	<0.0001
Waist circumference, cm	91.8±6.48	76.3±7.38	< 0.001	111.0±6.01	99.2±7.83	<0.0001
Hip circumference, cm	98.2±4.69	95.1±7.77	< 0.01	108.1±4.38	112.4±7.44	<0.001
Waist-to Hip ratio	0.93±0.05	0.80±0.05	<0.0001	1.03±0.06	0.88±0.06	<0.0001
BMI, kg/m²	25.4±2.08	22.9±3.2	<0.0001	31.6±2.15	30.6±3.24	ns
BMI -SDS	0.45±0.6	-0.12±0.93	< 0.001	1.83±0.39	1.57±0.50	0.01
BMI -Percentile	65.6±20.32	46.4±29.01	< 0.001	95.6±3.84	91.9±9.47	0.01
**Body composition**						
Seca						
Body weight, kg	81.9±7.95	62.7±9.67	<0.0001	102.1±8.20	84.3±10.4	<0.0001
Fat mass, kg	19.8±4.82	20.2±6.76	ns	32.9±5.27	36.5±7.05	0.01
Fat free mass, kg	62.03±5.2	42.5±4.87	<0.0001	69.2±5.48	47.8±4.66	<0.0001
Bod Pod						
Body weight, kg	81.9±7.93	62.7±9.69	<0.0001	102.1±8.20	84.2±10.4	<0.0001
Fat mass, kg	20.1±5.09	19.7±7.18	ns	36.2±6.15	38.2±7.78	ns
Fat free mass, kg	61.87±5.7	43.1±4.71	<0.0001	65.8±5.52	46.1±4.83	<0.0001
**Clinical chemistry**						
AST, U/l	24.9±9.63	22.3±6.05	ns	27.0±11.4	21.3±7.48	0.01
ALT, U/l	29.6±12.8	21.6±7.42	<0.0001	38.2±17.7	23.7±11.3	<0.0001
γGT, U/l	33.1±26.8	16.5±10.2	<0.0001	44.9±32.9	21.7±18.3	<0.0001
Sodium, mmol/l	141.0±1.94	141.3±1.69	ns	140.5±1.83	141.4±1.72	0.02
Potassium, mmol/l	4.50±0.37	4.43±0.33	ns	4.34±0.30	4.46±0.34	ns
Calcium, mmol/l	2.31±0.09	2.34±0.1	ns	2.30±0.09	2.29±0.10	ns
Phosphorus, mmol/l	3.31±0.40	3.71±0.46	<0.0001	3.23±0.40	3.57±0.38	<0.0001
Magnesium, , mmol/l	0.88±0.05	0.89±0.08	ns	0.87±0.05	0.87±0.05	ns
Cholesterol, mg/dl	220.3±28.5	228.8±48.6	ns	215.7±32.4	226.0±41.7	<0.0001
Triglycerides, mg/dl	113.9±58.6	84.6±42.6	< 0.01	172.9±80.4	120.1±44.3	<0.001
HDL-cholesterol, mg/dl	54.7±11.3	72.9±17.6	<0.0001	47.5±11.2	61.6±13.3	<0.0001

LDL-cholesterol, mg/dl	139.8±27.6	132.7±46.7	ns	143.0±33.6	144.9±36.9	ns
LDL/HDL	2.70±0.88	1.93±0.84	<0.0001	3.16±0.93	2.46±0.79	<0.001
Creatinine, mg/dl	0.91±0.13	0.75±0.10	<0.0001	0.88±0.11	0.72±0.09	<0.0001
Urea, mg/dl	34.8±7.46	29.9±7.72	< 0.01	6.98±1.32	28.1±6.17	0.02
Uric acid, mg/dl	6.18±1.12	4.31±0.84	<0.0001	6.98±1.32	5.09±1.05	<0.0001
Fasting blood glucose, mg/dl	84.5±7.37	80.2±8.0	< 0.01	98.6±33.6	83.7±8.6	<0.0001
Fasting insulin, µU/ml	3.72±2.45	3.50±3.75	ns	9.33±4.86	7.66±7.38	<0.01
TSH, µU/ml	1.50±0.83	1.25±0.59	ns	1.44±0.70	1.31±0.60	ns
FT3, pg/ml	3.29±0.83	3.07±0.38	<0.0001	3.39±0.24	3.12±0.30	<0.0001
FT4, ng/dl	1.15±0.32	1.11±0.17	ns	1.14±0.17	1.12±0.17	ns

Data are presented as mean ± standard deviation. *P*-values <0.05 were regarded as statistically significant. Mann-Whitney U test was used to test for significance between female and male participants. AST, aspartate aminotransferase; ALT, alanine aminotransferase; BMI, body mass index; γGT, gamma glutamyltransferase; HDL, high- density lipoprotein; LDL, low- density lipoprotein; MetS, metabolic syndrome; ns, not significant; TSH, thyroid-stimulating hormone; FT3, free thyroxine; FT4, free triiodothyronine.

Table 4. Study characteristics of older adults.

	male	female	p-value
**n**	81	79	
**Anthropometric**			
Age,y	78.1±2.51	78.2±2.98	ns
Height, cm	172.9±6.81	160.4±6.47	<0.0001
Waist circumference, cm	102.3±11.3	90.0±12.9	<0.0001
Hip circumference, cm	102.6±7.42	102.6±9.58	ns
Waist-to Hip ratio	1.0±0.08	0.88±0.09	<0.0001
Upper arm circumference, cm	30.0±2.63	28.5±3.27	<0.001
Calf circumference	36.9±2.59	35.8±2.76	ns
BMI, kg/m²	27.1±3.54	25.9±4.29	<0.05
BMI-SDS	0.69±0.91	0.72±1.02	ns
BMI-percentile	70.7±25.9	70.5±27.9	ns
**Body composition**			
**Seca**			
Body weight, kg	81.7±11.4	67.2±11.2	<0.0001
Fat mass, kg	25.7±7.25	28.9±8.18	<0.001
Fat free mass, kg	56.0±5.95	38.2±4.87	<0.0001
**BODPOD (only cohort in Freising)**			
Body weight, kg	79.7±10.9	65.0±11.4	<0.0001
Fat mass, kg	24.7±6.99	24.7±10.2	ns
Fat free mass, kg	54.9±6.29	40.3±3.06	<0.0001
**Laboratory analysis**			
AST, U/l	25.1±8.06	22.6±7.45	<0.05
ALT, U/l	22.9±8.88	20.3±7.40	<0.05
γGT, U/l	35.1±33.6	20.5±9.81	<0.0001
Sodium, mmol/l	141.6±2.20	142.1±2.36	<0.05
Potassium, mmol/l	4.67±0.55	4.60±0.47	ns
Calcium, mmol/l	2.32±0.10	2.40±0.11	<0.05
Phosphorus, mmol/l	2.96±0.44	3.47±0.48	<0.0001
Magnesium, , mmol/l	0.89±0.06	0.9±0.06	ns
Cholesterol, mg/dl	207.0±40.5	235.1±41.5	<0.0001
Triglycerides, mg/dl	106.2±42.4	108.5±44.5	ns

HDL-cholesterol, mg/dl	57.4±13.1	71.1±17.4	<0.0001
LDL-cholesterol, mg/dl	130.7±37.8	145.1±38.1	<0.05
LDL/HDL	2.39±0.86	2.80±0.79	ns
Creatinine, mg/dl	0.95±0.19	0.76±0.13	<0.0001
Urea, mg/dl	40.3±10.9	33.6±7.31	<0.0001
Uric acid, mg/dl	6.37±1.21	5.27±1.19	<0.0001
Fasting blood glucose, mg/dl	90.9±10.51	87.3±7.82	<0.05
Fasting insulin, µU/ml	5.09±3.80	5.01±4.28	ns
TSH, µU/ml	1.23±0.58	1.45±1.77	ns
FT3, pg/ml	3.23±0.38	2.95±0.31	<0.0001
FT4, ng/dl	1.15±0.18	1.17±0.20	ns

Data are presented as mean ± standard deviation. *P*-values <0.05 were regarded as statistically significant. Mann-Whitney U test was used to test for significance between female and male participants. AST, aspartate aminotransferase; ALT, alanine aminotransferase; BMI, body mass index, γGT, gamma glutamyltransferase; HDL, high- density lipoprotein; LDL, low- density lipoprotein; ns, not significant; TSH, thyroid-stimulating hormone; FT3, free thyroxine; FT4, free triiodothyronine.
